# Regional variation in Black infant mortality: The contribution of contextual factors

**DOI:** 10.1371/journal.pone.0237314

**Published:** 2020-08-11

**Authors:** Veni Kandasamy, Ashley H. Hirai, Jay S. Kaufman, Arthur R. James, Milton Kotelchuck

**Affiliations:** 1 Department of Population Family and Reproductive Health, Johns Hopkins Bloomberg School of Public Health, Baltimore, Maryland, United States of America; 2 Maternal and Child Health Bureau, Health Resources and Services Administration, Rockville, Maryland, United States of America; 3 Department of Epidemiology, Biostatistics & Occupational Health, McGill University, Montreal, Quebec, Canada; 4 Department of Obstetrics and Gynecology, Ohio State University, Columbus, Ohio, United States of America; 5 The Kirwan Institute for the Study of Race and Ethnicity, Ohio State University, Columbus, Ohio, United States of America; 6 Department of Pediatrics, Harvard Medical School/Massachusetts General Hospital, Boston, Massachusetts, United States of America; University of North Texas Health Science Center, UNITED STATES

## Abstract

**Background:**

Compared to other racial/ethnic groups, infant mortality rates (IMR) are persistently highestamong Black infants in the United States, yet there is considerable regional variation. We examined state and county-level contextual factors that may explain regional differences in Black IMR and identified potential strategies for improvement.

**Methods and findings:**

Black infant mortality data are from the Linked Birth/Infant Death files for 2009–2011. State and county contextual factors within social, economic, environmental, and health domains were compiled from various Census databases, the Food Environment Atlas, and the Area Health Resource File. Region was defined by the nine Census Divisions. We examined contextual associations with Black IMR using aggregated county-level Poisson regression with standard errors adjusted for clustering by state. Overall, Black IMR varied 1.5-fold across regions, ranging from 8.78 per 1,000 in New England to 13.77 per 1,000 in the Midwest. In adjusted models, the following factors were protective for Black IMR: higher state-level Black-White marriage rate (rate ratio (RR) per standard deviation (SD) increase = 0.81, 95% confidence interval (CI):0.70–0.95), higher state maternal and child health budget per capita (RR per SD = 0.96, 95% CI:0.92–0.99), and higher county-level Black index of concentration at the extremes (RR per SD = 0.85, 95% CI:0.81–0.90). Modeled variables accounted for 35% of the regional variation in Black IMR.

**Conclusions:**

These findings are broadly supportive of ongoing public policy efforts to enhance social integration across races, support health and social welfare program spending, and improve economic prosperity. Although contextual factors accounted for about a third of regional variation, further research is needed to more fully understand regional variation in Black IMR disparities.

## Introduction

In the United States, non-Hispanic Black (hereafter referred to as Black) infants have the highest rates of adverse birth outcomes, including preterm birth and infant mortality [1]. Black infants are more than twice as likely to die in the first year of life as compared to non-Hispanic White infants (hereafter referred to as White) [1]. Examination of geographic variation can be instructive in identifying contextual risk and protective factors. While overall and White infant mortality rates (IMR) are highest in the South, Black IMR tends to be highest in the Midwest and lowest in the West and Northeast [1].

Studies of regional variation may help us to better understand the heterogeneity of the Black birth experiences in the United States and may provide new insights into sources of excess Black IMR. Previous studies have focused on the sources of the Black-White disparity [2–5], which can be lower by virtue of high White versus low Black IMR [6], and limited in the provision of Black-specific contextual information as they are often dominated by the characteristics of the larger White community [5]. Studies that have examined Black infant mortality either alone or in addition to White infant mortality have suggested associations with various contextual factors within social, economic, environmental, and health domains but none have examined their contribution to regional variation [7–23]. Moreover, several studies have shown that the predictors of Black IMR are different than those of White IMR, and thus support a priority focus on the higher Black IMR [2, 4, 23, 24].

The current study focuses on regions to allow for a broader common historical and sociopolitical understanding of the Black experience in the United States, which may be masked in state-level analyses [6]. It remains unclear whether state and county contextual factors can explain broad regional patterns in Black IMR and help to identify risk and protective factors that may ameliorate higher rates in the Midwest and replicate or extend the lower rates observed in the West and Northeast [1]. Such information could inform regional multi-state action approaches to reducing infant mortality [25].

The aims of this study were:1) to examine regional variation in Black IMR and social, economic, environmental and health contextual factors;2) to identify which contextual factors are associated with Black IMR in multivariable models; and 3) to assess how much of the regional variation in Black IMR can be explained by these factors.

## Methods

### Data and measures

Birth and mortality data for Black infants were obtained from the National Center for Health Statistics’ 2009–2011 linked birth/infant death files with county and state identifiers obtained by request from the National Association of Public Health Statistics and Information Systems [26]. The selected data years center upon 2010 given various covariates drawn from the 2010 Census. Regional units were defined by Census Divisions (hereafter referred to as regions). These nine regions are based on the Census Bureau’s framework of large units that are relatively similar in terms of population characteristics, economic, and historical development [27].

We constructed an aggregated county-level dataset with a count of Black births, Black infant deaths, and linked county and state-level characteristics. We limited the analysis to counties with complete data on covariates (2450 counties of the 2708 counties with Black births), which resulted in a loss of 29 deaths (retained 99.9% of deaths) and 3209 births (retained 99.8% of births). Ethical approval was not needed as fully anonymized vital records data are publicly available through a data use agreement with the National Center for Health Statistics.

Various state and county-level factors in the social, economic, environment and health domains were compiled from Census databases, the Food Environment Atlas, and the Area Health Resource File [28]. We used Black-specific data wherever possible. Of approximately 75variables initially compiled (S1 Appendix), 16 were selected for a final model based on prior literature on adverse birth outcomes or health disparities and consideration for both literature support and strength of association when deciding between collinear variables. S2 Appendix includes the level (state or county), year, data source, and calculation details for each of the variables included in the final model.

The final social variables in the model were percent Non-Hispanic Black population (state) [23], Black-White marriage rate (state) [21, 29], a hypersegregation index (county) [22–24, 30–32], Black incarceration rate (state) [7, 13, 33], and percent of voting age population casting votes (state) [11]. The Black-White marriage rate, calculated as the percentage of married Black individuals with a White spouse, was selected as a contextual measure of social integration [29, 34, 35]. The hyper segregation index was calculated based on dissimilarity (a measure of evenness, which is the proportion of Black residents required to change census tracts to get an even distribution of minorities in a county) and isolation (a measure of exposure, which is the extent to which members of the Black (minority) population are exposed only to each other, rather than to members of the White (majority) population [36, 37]. Other literature has indicated that looking at these two (of five) conventional measures of segregation is sufficient to assess hyper segregation [38, 39].

Economic variables included the Index of Concentration at Extremes (ICE) based on Black household income (county) [18, 40–42], and Black unemployment rate (county) [43]. The Black ICE is a measure of spatial economic polarization and ranges from -1 (all of the population is among the most deprived group; <20% percentile of household income) to 1 (all of the population is among the most privileged group; >80% percentile of household income) [19, 20]. The universe for the Black ICE is all Black households.

Environmental variables included daily fine particulate matter in micrograms per cubic meter (PM2.5)(county) [44, 45], grocery stores per 1,000 population (county) [46, 47], housing unit vacancy percent (county) [7, 10, 48], and National Center of Health Statistics’ 2006 urban/rural classification (county) [49, 50].

Health variables included Medicaid eligibility for pregnant women based on percent of federal poverty level (state) [17, 51, 52], maternal and child health (MCH) budget per capita (state) [53, 54], nurse midwives per 100,000 women aged 15–44 (county) [55–57], obstetricians/gynecologists per 100,000 women aged 15–44 (county) [14, 58–60], and percent of uninsured women aged 18–44 (county) [2, 7, 61].

### Statistical analysis

Black IMR and contextual covariates were descriptively examined by region (Aim 1) using overall totals (sum of deaths and births) for IMR and means or proportions for covariates (weighted by births) to represent average contextual characteristic per Black birth in each region. Unadjusted and adjusted associations (Aim 2) between contextual characteristics and Black IMR were examined using aggregated county-level Poisson regression of deaths over birth counts with standard errors adjusted for clustering by state through generalized estimating equations [62]. This type of aggregated analysis offers a more efficient data structure with identical results as those obtained by linking individual observations to county or state characteristics [63]. Each county was linked to various county and state covariates. Relative rate ratios (RR) and absolute rate differences were estimated through average marginal prediction contrasts [64] and calculated relative to a reference for categorical variables and per standard deviation (SD)and mean plus SD for continuous variables, respectively. We compared unadjusted and model-adjusted region-level IMRs to estimate regional variation before and after adjustment and the proportion attributable to model characteristics (Aim 3). We used simple variance formulas for fixed differences rather than a random effects approach given the small number of regional units for reliable random effects variance estimation [62, 65]. All analyses were performed using SAS 9.4 (SAS Institute Inc., Cary, NC) and SAS-callable SUDAAN 11.0.1 (RTI International, Research Triangle Park, NC).

## Results

### Regional variation in Black IMR

**Fig 1:**
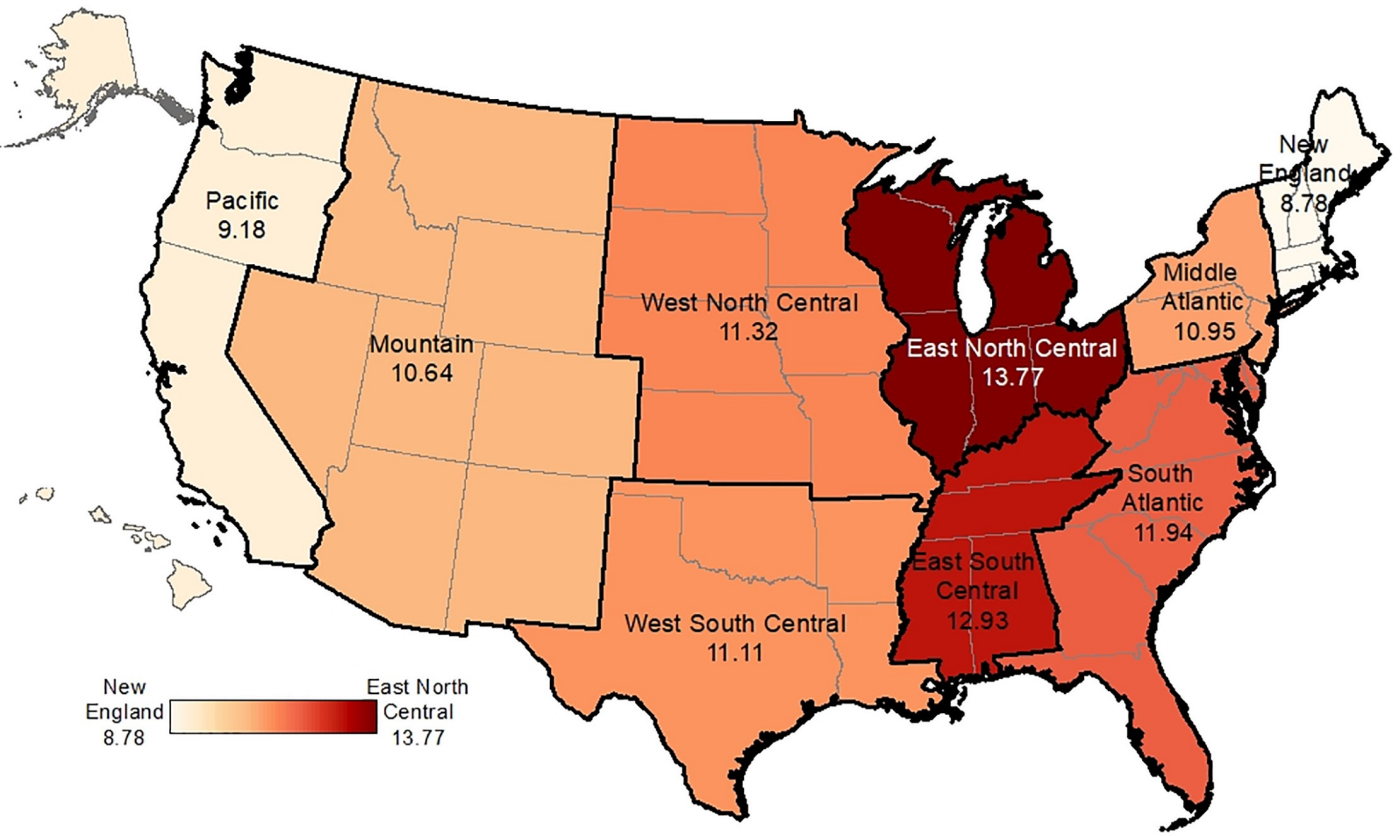
Black infant mortality rates per 1,000 live births by region, 2009–2011. The overall Black IMR was 11.78 per 1,000, which varied 1.5-fold across regions ranging from 8.78 deaths per 1,000 in New England to 13.77 deaths per 1,000 in the East North Central region. The basemap (shapefile) was retrieved from Census TIGER/Line https://www.census.gov/geographies/mapping-files/time-series/geo/tiger-line-file.html and the color scheme is an option within ESRI ArcGIS Desktop https://www.esri.com/en-us/arcgis/products/arcgis-desktop/overview.

The overall Black IMR was 11.78 per 1,000, which varied 1.5-fold across regions (Table 1, Fig 1). Black IMR was lowest in New England and Pacific regions (8.78 and 9.19 per 1,000) and highest in East North Central and East South Central (13.77 and 12.92 per 1,000, respectively). Black births were not evenly distributed across the regions, with fewer births in areas where IMR was lower, ranging from about 2% in New England and the Pacific to over 30% in the South Atlantic.

**Table 1:** Black births, deaths and infant mortality rate by region, 2009–2011.

Variable	Overall	New England (1)	Middle Atlantic (2)	East North Central (3)	West North Central (4)	South Atlantic (5)	East South Central (6)	West South Central (7)	Mountain (8)	Pacific (9)
Deaths	20959	359	2545	3740	852	7118	2272	2701	388	985
Births	1778528	40867	232436	271546	75263	595902	175771	243045	36451	107246
Black IMR	11.78	8.78	10.95	13.77	11.32	11.94	12.93	11.11	10.64	9.18
Percent of Deaths by Region		1.71	12.14	17.84	4.07	33.96	10.84	12.89	1.85	4.70
Percent of Births by Region		2.30	13.07	15.27	4.23	33.51	9.88	13.67	2.05	6.03

Abbreviations: IMR, infant mortality rate.

### Regional variation in contextual factors

All covariates differed by region (Table 2). Variables with the largest variation by region, with coefficients of variation exceeding 50%, included the state-level non-Hispanic Black percentage, the state-level Black-White marriage rate, county hypersegregation, rurality, and the annual state MCH budget per capita. The average state non-Hispanic Black percentage was 17.3% and ranged from approximately 5% in Mountain and Pacific Regions to approximately 25% in the South Atlantic and East South Central. The average state percentage of Black marriages to Whites was 6.0% ranging from 3.3% in East South Central to 16.9% in the Mountain region. Overall, about 20% of Black births occurred in hypersegregated counties with high dissimilarity and isolation (Table 2). The East North Central and Middle Atlantic regions had the highest hypersegregation, where approximately 40–50% of births occurred in hypersegregated counties. By contrast, there were no hypersegregated counties in New England, Mountain, and Pacific regions. Overall, 9.9% of Black births occurred in rural counties, which ranged from under 2% in New England, Middle Atlantic, and East North Central regions to 28.6% in East South Central. The average annual state MCH budget per capita was about $16, ranging from under $3 in West North Central and Mountain regions to nearly $60 in the Pacific region.

**Table 2:** Descriptive statistics and variation by region, 2009–2011.

			Mean (SD) by Region, weighted by births								
Variable	Mean (SD) Overall	CV by Region	New England (1)	Middle Atlantic (2)	East North Central (3)	West North Central (4)	South Atlantic (5)	East South Central (6)	West South Central (7)	Mountain (8)	Pacific (9)
***Social***											
Percent Non-Hispanic Black Population	17.31 (9.21)	56%	6.86 (2.15)	12.96 (1.66)	12.44 (2.45)	7.83 (3.47)	23.65 (7.36)	24.96 (9.55)	17.68 (9.39)	4.64 (2.15)	5.4 (1.02)
Black-White marriage rate	5.97 (4.44)	54%	12.35 (7.6)	5.55 (1.49)	7.02 (2.11)	13.69 (6.44)	3.94 (1.57)	3.33 (2.55)	4.2 (2.21)	16.86 (6.84)	12.25 (5.18)
Segregation Index											
Both dissimilarity and isolation <0.6	64.70	31%	76.20	40.43	36.79	73.55	71.78	54.82	83.36	99.99	99.93
One index > = 0.6	14.06	78%	23.80	17.89	11.61	0.48	16.41	23.42	11.43	0.01	0.07
Both indices > = 0.6	21.24	103%	0.00	41.67	51.60	25.97	11.81	21.76	5.21	0.00	0.00
Black incarceration rate (per 100,000 in adult population)	2324.31 (604.41)	18%	1815.56 (357.73)	2167.93 (687.82)	2412.82 (511.7)	2558.89 (465.89)	2073.73 (527.93)	1996.64 (493.75)	2841.42 (270.29)	3094.99 (407.17)	2963.89 (216.53)
Percent of voting age population casting votes for 2008 presidential electors	64 (4.48)	4%	67.32 (0.87)	60.91 (2.25)	65.03 (2.99)	68.33 (4.22)	65.98 (2.45)	62.72 (5.48)	60.31 (6.61)	61.95 (4.13)	63.93 (1.28)
***Economic***											
Black Index of Concentration at the Extremes	-0.26 (0.17)	32%	-0.16 (0.1)	-0.19 (0.17)	-0.34 (0.11)	-0.34 (0.14)	-0.23 (0.19)	-0.38 (0.11)	-0.29 (0.15)	-0.2 (0.09)	-0.15 (0.11)
Black Civilian Unemployment Rate	16.27 (4.27)	12%	15.45 (3.13)	15.36 (2.89)	20.61 (4.19)	17.34 (4.54)	15.56 (3.77)	16.45 (4.54)	13.44 (3.22)	15.2 (3.44)	17.19 (2.94)
***Environment***											
Daily Fine Particulate Matter	11.88 (1.41)	11%	10.91 (0.11)	11.47 (0.72)	12.97 (0.33)	11.52 (1.43)	12.51 (0.64)	12.73 (0.53)	10.72 (1.03)	11.56 (1.79)	8.45 (1.3)
Grocery Stores per 1,000 population in 2009	0.22 (0.12)	36%	0.21 (0.04)	0.42 (0.2)	0.21 (0.05)	0.18 (0.07)	0.2 (0.06)	0.18 (0.06)	0.18 (0.08)	0.13 (0.02)	0.21 (0.04)
Housing Unit Vacancy Percent	10.6 (4.44)	17%	7.78 (4.24)	8.19 (3.29)	10.41 (2.57)	9.4 (4.3)	12.07 (5.29)	11.64 (3.21)	10.71 (4.25)	11.92 (4.31)	7.68 (2.69)
Rural Urban Classification											
Large urban counties	64.34	23%	62.69	86.98	73.98	67.64	58.82	36.76	53.68	77.99	84.53
Small and medium urban counties	25.75	33%	36.04	11.88	24.21	24.19	28.80	34.63	31.56	18.06	14.79
Rural counties	9.92	108%	1.27	1.14	1.81	8.17	12.38	28.61	14.76	3.95	0.68
***Health***											
Medicaid eligibility for pregnant women as % FPL	192.19 (29.6)	11%	216.44 (25.2)	192.7 (7.5)	203.99 (28.33)	211.57 (50.5)	193.41 (33.66)	168.61 (24.16)	187.4 (10.49)	142.91 (20.46)	197.77 (5.33)
Maternal and child health budget per person/capita	15.84 (17.65)	110%	6.19 (4.07)	28.93 (20.41)	10.67 (6.84)	2.53 (0.59)	15.22 (11.97)	9.18 (6.86)	4.74 (2.47)	2.98 (3.6)	57.55 (22.3)
Certified Nurse Midwives per 100,000 women ages 15–44	19.44 (14.73)	41%	39.41 (13.33)	25.52 (10.22)	20.07 (10.05)	19.28 (15.86)	22.92 (16.25)	10.77 (12.58)	7.81 (7.82)	21.95 (18.26)	17.55 (12.61)
Obstetricians/Gynecologists per 100,000 women ages 15–44	67.52 (33.83)	14%	87.03 (27.5)	71.8 (34.59)	73.66 (26.53)	74.07 (34.11)	65.62 (37.67)	65 (35.83)	64.59 (33.48)	55.72 (20.28)	56.03 (17.05)
Percent Uninsured Females 18–44 years	22.36 (7.56)	29%	8.53 (4.45)	16.91 (4.23)	18.18 (3.62)	17.57 (6.2)	24.05 (7.35)	22.61 (4.81)	30.75 (6.36)	24.43 (4.31)	23.82 (5.3)

SD = Standard Deviation CV = Coefficient of Variation FPL = Federal Poverty Level.

### Contextual associations with Black IMR

The following factors were associated with Black IMR in unadjusted models: Black-White marriage rate, Black ICE, Black unemployment, daily fine particulate matter, housing vacancy, rural/urban classification, and MCH budget per capita (Table 3). After adjustment, only the Black-White marriage rate, Black ICE, and MCH budget per capita remained associated. The Black-White marriage rate and Black ICE also had the largest adjusted associations. For every one SD increase in the state percentage of married Black individuals with White spouses (SD: 14%, not shown in tables), the Black IMR decreased by 19% (adjusted RR: 0.81, 95% confidence interval (CI):0.70–0.95), corresponding to two fewer deaths per 1,000 births. For every one SD increase in the county Black ICE(SD: 0.3, not shown in tables), the Black IMR was 15% lower (adjusted RR: 0.85, 95% CI:0.81–0.90), corresponding to 1.7 fewer deaths per 1,000 births. The adjusted RR of 0.96 (95% CI:0.92–0.99) for the state MCH budget per capita indicates that for every one SD increase (SD: $13 per capita, not shown in tables), the Black IMR decreased by 4%.

**Table 3:** Unadjusted and adjusted associations (rate ratio and rate difference) between contextual factors and the Black infant mortality rate, 2009–2011.

	Unadjusted			Adjusted		
Variable	Rate Ratio (RR) (per SD continuous variables) with 95% CI	Rate Difference (RD) (mean + 1SD) per 1,000 with 95% CI	P-value Wald F	Rate Ratio (RR) (per SD continuous variables) with 95% CI	Rate Difference (RD) (mean + 1SD) per 1,000 with 95% CI	P-value Wald F
***Social***						
Percent Non-Hispanic Black Population	1.04 (0.98, 1.1)	0.44 (-0.05, 0.93)	0.18	0.95 (0.88, 1.04)	-0.56 (-1.22, 0.10)	0.25
Black-White marriage rate	0.88 (0.77, 1.00)	-1.34 (-2.56, -0.12)	0.05	0.81 (0.70, 0.95)	-1.97 (-3.09, -0.85)	0.01
Segregation Index			0.23			0.64
Both dissimilarity and isolation <0.6	ref	ref		ref	ref	
One index > = 0.6	1.08 (0.97, 1.21)	0.93 (-0.40, 2.26)		1.03 (0.93, 1.13)	0.33 (-0.77, 1.43)	
Both indices > = 0.6	1.07 (0.95, 1.20)	0.79 (-0.58, 2.16)		1.03 (0.96, 1.12)	0.39 (-0.49, 1.27)	
Black incarceration rate (per 100,000 in adult population)	0.98 (0.90, 1.07)	-0.24 (-0.92, 0.44)	0.63	0.97 (0.90, 1.04)	-0.37 (-0.93, 0.19)	0.34
Percent of voting age population casting votes for 2008 presidential electors	1.04 (0.99, 1.09)	0.46 (-0.04, 0.96)	0.15	1.02 (0.97, 1.07)	0.20 (-0.46, 0.86)	0.47
***Economic***						
Black Index of Concentration at the Extremes	0.84 (0.79, 0.90)	-1.93 (-2.42, -1.44)	0.00	0.85 (0.81, 0.90)	-1.66 (-2.2, -1.12)	0.00
Black Civilian Unemployment Rate	1.22 (1.13, 1.31)	2.55 (1.94, 3.16)	0.00	1.00 (0.92, 1.08)	-0.05 (-0.67, 0.57)	0.92
***Environment***						
Daily Fine Particulate Matter	1.08 (1.04, 1.13)	0.99 (0.49, 1.49)	0.00	0.98 (0.92, 1.04)	-0.28 (-0.95, 0.39)	0.42
Grocery Stores per 1,000 population in 2009	0.96 (0.90, 1.02)	-0.48 (-1.02, 0.06)	0.19	0.96 (0.91, 1.01)	-0.49 (-1.13, 0.15)	0.11
Housing Unit Vacancy Percent	1.09 (1.03, 1.15)	1.08 (0.39, 1.77)	0.01	1.01 (0.97, 1.06)	0.15 (-0.44, 0.74)	0.52
Rural Urban Classification			0.00			0.22
Large urban counties	ref	ref		ref	ref	
Small and medium urban counties	1.12 (1.06, 1.20)	1.40 (0.69, 2.11)		1.05 (0.98, 1.13)	0.60 (-0.14, 1.34)	
Rural counties	1.10 (1.00, 1.21)	1.11 (0.01, 2.21)		1.01 (0.92, 1.11)	0.09 (-0.95, 1.13)	
***Health***						
Medicaid eligibility for pregnant women as % FPL	0.98 (0.93, 1.03)	-0.23 (-0.77, 0.31)	0.43	1.01 (0.96, 1.06)	0.08 (-0.52, 0.68)	0.78
Maternal and child health budget per person/capita	0.95 (0.93, 0.97)	-0.56 (-0.91, -0.21)	0.00	0.96 (0.92, 0.99)	-0.51 (-1.07, 0.05)	0.02
Certified Nurse Midwives per 100,000 women ages 15–44	0.97 (0.91, 1.03)	-0.38 (-0.93, 0.17)	0.29	0.97 (0.93, 1.00)	-0.38 (-0.95, 0.19)	0.05
Obstetricians/Gynecologists per 100,000 women ages 15–44	1.02 (0.99, 1.05)	0.22 (-0.26, 0.70)	0.26	1.02 (0.99, 1.06)	0.25 (-0.28, 0.78)	0.21
Percent Uninsured Females 18–44 years	0.98 (0.94, 1.03)	-0.22 (-0.68, 0.24)	0.41	0.95 (0.91, 1.00)	-0.52 (-1.09, 0.05)	0.08
***Geographic Region***						
Census Division			0.00			0.06
New England (1)	0.64 (0.48, 0.84)	-5.00 (-7.39, -2.61)		0.67 (0.49, 0.90)	-4.30 (-6.93, -1.67)	
Middle Atlantic (2)	0.79 (0.69, 0.92)	-2.80 (-4.37, -1.23)		0.95 (0.85, 1.06)	-0.60 (-1.93, 0.73)	
East North Central (3)	ref	ref		ref	ref	
West North Central (4)	0.82 (0.69, 0.98)	-2.50 (-4.48, -0.52)		0.83 (0.70, 0.98)	-2.10 (-3.94, -0.26)	
South Atlantic (5)	0.87 (0.8, 0.94)	-1.80 (-2.70, -0.90)		0.95 (0.86, 1.05)	-0.60 (-1.80, 0.60)	
East South Central (6)	0.94 (0.88, 1.00)	-0.80 (-1.58, -0.02)		0.91 (0.81, 1.03)	-1.10 (-2.57, 0.37)	
West South Central (7)	0.81 (0.75, 0.87)	-2.70 (-3.54, -1.86)		0.83 (0.70, 0.97)	-2.20 (-4.00, -0.40)	
Mountain (8)	0.77 (0.67, 0.89)	-3.10 (-4.57, -1.63)		0.95 (0.75, 1.20)	-0.70 (-3.50, 2.10)	
Pacific (9)	0.67 (0.61, 0.73)	-4.60 (-5.44, -3.76)		0.92 (0.68, 1.24)	-1.10 (-4.53, 2.33)	

SD = standard deviation, FPL = federal Poverty Level, CI = confidence interval.

### Regional variation explained by contextual factors

The regional variance in Black IMR decreased from 2.28 before adjustment to 1.48 after adjustment, representing a 35% reduction. (Table 4) There were five regions that had sizeable changes between unadjusted and adjusted IMRs, with differences exceeding 1 death per 1,000. The East North Central and East South Central regions had the highest unadjusted IMRs and had the largest decreases in IMR after adjustment. The Pacific, Mountain, and Middle Atlantic regions had large increases in Black IMR after adjustment. The South Atlantic and New England regions had the smallest changes in Black IMR after adjustment.

**Table 4:** Unadjusted and adjusted Black infant mortality rate by region and variance explained, 2009–2011.

Region	Unadjusted IMR	Adjusted IMR	Percent change	Absolute change
New England (1)	8.78 (6.67, 11.54)	8.46 (6.26, 11.44)	-3.64%	-0.32
Middle Atlantic (2)	10.95 (9.52, 12.59)	12.08 (11.12, 13.12)	10.32%	1.13
East North Central (3)	13.77 (13.3, 14.25)	12.72 (11.61, 13.93)	-7.63%	-1.05
West North Central (4)	11.32 (9.5, 13.49)	10.57 (9.15, 12.21)	-6.63%	-0.75
South Atlantic (5)	11.94 (11.17, 12.77)	12.11 (11.12, 13.18)	1.42%	0.17
East South Central (6)	12.92 (12.29, 13.59)	11.6 (10.53, 12.78)	-10.22%	-1.32
West South Central (7)	11.11 (10.43, 11.85)	10.52 (9.38, 11.81)	-5.31%	-0.59
Mountain (8)	10.63 (9.3, 12.14)	12.06 (9.92, 14.65)	13.45%	1.43
Pacific (9)	9.19 (8.52, 9.92)	11.66 (8.87, 15.34)	26.88%	2.47
			**Proportion of variance explained**	
*Variance*	2.28	1.48	-35%	

IMR = Infant mortality rate.

## Discussion

The results from this state and county contextual examination of Black infant mortality revealed that the Black-White marriage rate, MCH budget per capita, and Black ICE explained approximately one-third of the regional variation in the Black IMR. The East North Central (the Midwest) and East South Central regions had the highest unadjusted Black IMR and had the largest decreases in Black IMR after adjustment, indicating that modeled covariates captured some of the contextual factors associated with their regional disadvantage. The Pacific, Mountain, and Middle Atlantic regions all had large increases in Black IMR after adjustment, indicating that these regions had certain advantages explained by the model. However, the New England IMR advantage was not explained by model covariates. Each of the three protective factors we identified represent distinct potential policy avenues to improve social integration, public health spending, and household income. These factors may inform state and local efforts to reduce Black IMR as well as potential multi-state regional action collaboratives [25].

In adjusted models, the state-level Black-White marriage rate was associated with the greatest relative risk reduction in Black IMR. The Black-White marriage rate can be viewed as a measure of inter-racial social intimacy and integration, where the majority and minority groups view each other with greater equality, familiarity, and trust [29]. Structural and social conditions, including residential segregation, interpersonal discrimination, and anti-miscegenation laws, have led to a history of prohibition against interracial marriage [34]. Thus, this measure can also be viewed more broadly as a reflection of racial intergroup acceptance, and the converse of multiple dimensions of racism—structural, cultural, and interpersonal. Changes in interracial marriage trends by region over time may reflect a counter measure of “latent” racism [35]. While other related contextual measures of racism, such as residential segregation or Black-White inequalities in education, income, and employment, have been associated with adverse Black birth outcomes [4, 23, 41, 66–68], the state-level Black-White marriage rate could be considered a novel positive indicator of integration in general. Although county-level hypersegregation was not associated with Black IMR before or after adjustment, more studies indicate an association with birth outcomes when measured at the census tract or Metropolitan Statistical Area (MSA) level [23, 69, 70].

It has been posited that increasing social expenditures, such as state MCH budgetary expenditures, may reduce IMR and racial disparities in IMR [71]. State MCH expenditures are part of the Title V MCH Block Grant with an expressed mission that includes reducing infant mortality and ensuring access to quality health services for vulnerable populations with lower incomes. Our novel finding associating higher state MCH expenditures with lower Black infant mortality is consistent with other evidence at both state and local-levels that have linked public health expenditures with infant health and birth outcomes [16, 53, 54, 72–76]. A recent Florida study that examined county-level MCH expenditures and infant mortality found that greater spending was associated with reductions in IMR, especially for the Black population, which may have a greater need and benefit from funded public health and social services [74]. Importantly, many prior studies controlled for state or county fixed effects to account for other unobserved differences that may be associated with increased public health spending [16, 54, 74, 76].

Higher Black ICE was also associated with lower Black IMR, consistent with a growing body of literature connecting ICE with birth outcomes at both county and city levels [18, 19, 41]. Unlike other economic measures, such as poverty, household income, or income inequality, ICE captures both privilege and deprivation and the relative balance between the two [20, 77]. Another version of ICE that captures racial economic polarization [19] was examined but was not associated with Black IMR after adjustment, perhaps due to other related measures such as Black-White marriage rates.

### Limitations

A variety of other social, economic, environmental, and health characteristics were not associated with regional Black IMR in the mutually adjusted model, which may reflect high correlation between measures, and a combination of lack of content association and poor measurement. Several variables could only be measured at the state rather than county level (e.g. incarceration rates) and some were not measured specifically within the Black-community (e.g. voting percentages). This study posits substantial room for further development and contextual exploration of race-specific measures assessed at more granular geographic levels. Despite their persistent associations with the outcome, state MCH expenditures and the Black-White marriage rate also have limitations. State MCH expenditures were not specific to those related to women’s or infant health versus children’s health investments. For Black-White marriage, the restriction to married couples in an era of declining marriage [78] may reflect a measure of racial integration among higher socioeconomic status groups. It is also sensitive to same race partner availability, although we controlled for the percent of non-Hispanic Black population. In addition, state-level Black-White marriage may reflect mixed race on an individual-level or paternal White race, which is associated with better birth outcomes compared to single race Black and paternal Black race. While evidence suggests that this reflects social rather than genetic contributions [21], future research could explore the association of state-level Black-White marriage with birth outcomes among single race Black mothers and fathers. All states adopted the 2003 birth certificate revision that distinguishes single and multiple race as of 2016 with the first year of complete 2017 infant mortality follow-up released in 2019. Although more recent infant mortality data are available, we relied on the 2010 Census for several variables and IMR has not changed substantially; thus, results are still relevant.

There are also broader limitations of the study’s overall approach. The cross-sectional design limits assessment of causal relations. Only associations can be documented, which are subject to confounding by unmeasured policies and characteristics. Some conceptual contextual measures (e.g. racism, segregation, etc.) are difficult to operationalize and therefore may have been imprecisely measured. Variables may also operate at a different or more granular level than available in national vital records (e.g. neighborhood level). Further, individual characteristics were not included in the ecological models, though they are likely to mediate the contextual effects that were of primary interest. Finally, aggregating geographic units (counties into states into regions) can create a modifiable areal unit problem in which results depend on the boundaries and definitions applied. Although counties and states hold specific administrative and policy functions with standard boundaries, we selected the Census Bureau groupings of states into regions based on similar population characteristics, economic, and historical development [27], which also appeared to fit Black IMR patterns.

### Implications

Conceptually, our approach addresses the contextual contributions to poor birth outcomes rather than individual behaviors, personal, health or genetic characteristics. Further longitudinal research to examine changes in contextual factors in relation to changes in outcomes would significantly strengthen inference. Additional research could also enhance and refine measurement at multiple scales to explain more of the variation in Black IMR and examine other adverse birth outcomes.

Programs to improve racial integration and economic opportunity may require policy and programmatic interventions, preferentially targeted to populations experiencing the worst outcomes. Placed-based transformations, such as Best Babies Zones, free college education, and paid parental leave, reflect efforts to address income inequality and structural racism [79–83]. The concentration of poverty within the Black community, a product of discrimination and unequal opportunity, remains a very serious problem for optimal reproductive health [84].

## Conclusions

This study is part of the continuing efforts to understand the roots of the unacceptably high Black IMR in the United States. Three contextual factors, state Black-White marriage rate, county Black ICE, and state MCH budget, were strongly and independently associated with Black IMR, accounting for over a third of regional variation. These associations encourage continuing efforts to improve social integration across racial groups, increase public spending on MCH health and services programs, and address income disparities that together may influence Black IMR. Additional contextual research is needed to advance knowledge of causation and understanding of regional and other variations in poor reproductive outcomes within the heterogeneous Black community.

## Supporting information

S1 Appendix Variables and data sources for 75 considered variables. (DOCX)

S2 Appendix Data source and notes for all 16 variables in final model. (DOCX)
