# Supplementary material for: Regional variation in Black infant mortality: The contribution of contextual factors
Source: PLoS One. 2020 Aug 11;15(8):e0237314. doi: 10.1371/journal.pone.0237314 (PMC7418975; doi:10.1371/journal.pone.0237314)
Supplement: S1 Appendix — (DOCX) [file pone.0237314.s001.docx]

S1 Appendix: Variables and Data Sources for 75 Considered Variables

| **County-Level Variables** | **Source** |
| --- | --- |
| Non-Hispanic Black Percentage | 2010 Census Summary File (SF) 1 |
| Gini Index | 2008-2012 American Community Survey (ACS) 5-Year Estimates. Gini Index of Income Inequality |
| Index of Dissimilarity | 2010 Census SF 1 |
| Index of Isolation | 2010 Census SF 1 |
| Segregation Index | 2010 Census SF 1 |
| Violent Crime per 1,000Population | USA Counties Database. Department of Justice (DOJ)-Federal Bureau of Investigation (FBI), 2008. (http://www.census.gov/support/USACdataDownloads.html) |
| Incarceration Rate Per 1,000 Population | 2010 Census SF 1 |
| Black Incarceration Rate (per 100,000 in Adult Population) | 2010 Census SF 1 (calculated by prisonpolicy.org) |
| Food Environment Index | County Health Rankings, 2014 data; USDA Food Environment Atlas, Map the Meal Gap from Feeding America; http://www.countyhealthrankings.org/app/maryland/2015/measure/factors/133/description |
| Food Environment Index Quartile | County Health Rankings, 2014 data; USDA Food Environment Atlas, Map the Meal Gap from Feeding America; http://www.countyhealthrankings.org/app/maryland/2015/measure/factors/133/description |
| Primary Care Providers per 100,000Population | Area Health Resource File (AHRF)-Health Professionals-Primary Care physicians, 2010 (data from American Medical Association Physician Masterfile) |
| Obstetricians/Gynecologists per 100,000 Women ages 15-44 | AHRF-Health Professionals-MDs by Specialty (Surgical), 2010 (data from American Medical Association Physician Masterfile) |
| HPSA Primary Care Shortage Designation | AHRF-Codes & Classifications, 1 category: HPSA Code - Primary Care (2010) (data from Bureau of Primary Health Care) |
| Number of CHCs per 100,000 Population | AHRF-Health Facilities-Providers of Services, 1 category: # Fed Qualified Health Centers data for 2010 (data from the Centers for Medicare and Medicaid Services’ (CMS)) |
| Certified Nurse Midwives per 100,000 Women ages 15-44 | AHRF-Health Professionals-Other Health Professionals, for 2011 (data from CMS National Provider Identification (NPI) File) |
| Daily Fine Particulate Matter | AHRF-Environment-Daily Fine Particulate Matter (2010) (Data from the Centers for Disease Control and Prevention Wonder online database) |
| Grocery Stores | 2012 U.S. Department of Agriculture (USDA) Economic Research Service (ERS) Data Download |
| Grocery Stores per 1,000 Population | 2012 USDA ERS Data Download |
| Convenience Stores | 2012 USDA ERS Data Download |
| Convenience Stores per 1,000 Population | 2012 USDA ERS Data Download |
| Fast Food Restaurants | 2012 USDA ERS Data Download |
| Fast food restaurants per 1,000 Population | 2012 USDA ERS Data Download |
| Retail Food Environment Index (RFEI) | 2012 USDA ERS Data Download |
| NCHS Rural Urban Classification | NCHS Rural Urban Classification from CDC (http://www.cdc.gov/nchs/data_access/urban_rural.htm;) |
| Total Civilian Unemployment Rate | AHRF-Population-ACS Employment. Data from 2008-2012, ACS Summary File, U.S. Census Bureau |
| Total Civilian Black Unemployment Rate | AHRF-Population-ACS Employment. Data from 2008-2012, ACS Summary File, U.S. Census Bureau |
| Low Employment | AHRF-Codes & Classifications-Low Employment Code (2004) (2004 County Typology Codes are from USDA ERS) |
| Percent of Persons 25+ (25 and older)with less than (<) High School (HS) Diploma | AHRF-Population-ACS Education. Data from ACS Summary File, U.S. Census Bureau. |
| Percent of Black Persons 25+ with <HS Diploma | AHRF-Population-ACS Education. Data from ACS Summary File, U.S. Census Bureau. |
| Percent of Persons 25+ with HS DiplomaOr More | AHRF-Population-ACS Education. Data from ACS Summary File, U.S. Census Bureau. |
| Percent of Black Persons 25+ with High School DiplomaOr More | AHRF-Population-ACS Education. Data from ACS Summary File, U.S. Census Bureau. |
| Percent of Persons 25+ with 4+ Years College | AHRF-Population-ACS Education. Data from ACS Summary File, U.S. Census Bureau. |
| Percent of Black Persons 25+ with 4+ Years College | AHRF-Population-ACS Education. Data from ACS Summary File, U.S. Census Bureau. |
| Low Education | AHRF-Codes & Classifications-Low Education Code (2004) (data from USDA ERS) |
| Percent of Uninsured Females <18 | 2008-2012 ACS 5-year Estimates (2012). Health Insurance Coverage Status |
| Percent of Uninsured Females 18-64 | 2008-2012 ACS 5-year Estimates (2012). Health Insurance Coverage Status |
| Percent of Uninsured Females 18-44 | 2008-2012 ACS 5-year Estimates (2012). Health Insurance Coverage Status |
| Percent of Occupied Housing Units | 2010 Census SF 1. Profile of General Population and Housing Characteristics: 2010 |
| Percent of Owner-Occupied Housing Units | 2011 Census SF 1. Profile of General Population and Housing Characteristics: 2010 |
| Percent of Unoccupied Housing Units | 2012 Census SF 1. Profile of General Population and Housing Characteristics: 2010 |
| Housing Stress | AHRF-Codes & Classifications-Housing Stress Code (2004) (data from USDA ERS) |
| Median Household Income | 2008-2012 ACS 5-Year Estimates. Median Family Income in the Past 12 Months |
| Median BlackHousehold Income | 2008-2012 ACS 5-Year Estimates. Median Family Income in the Past 12 Months |
| Total Index of Concentration at the Extremes (ICE) | AHRF-Population-ACS Income Statistics. 2008-2012, ACS Summary File, U.S. Census Bureau |
| Black ICE | AHRF-Population-ACS Income Statistics. 2008-2012, ACS Summary File, U.S. Census Bureau |
| Percent of Persons in Deep Poverty | 2008-2012 ACS 5-Year Estimates. % Persons in Deep Poverty |
| Percent of Children < 18 in Deep Poverty | 2008-2012 ACS 5-Year Estimates. % Children <18 in Deep Poverty |
| Persistent Poverty | USDA ERS (http://www.ers.usda.gov/topics/rural-economy-population/rural-poverty-well-being/geography-of-poverty.aspx) |
| Percent of Persons in Poverty | 2008-2012 ACS 5-year Estimates. Poverty Status In the Past 12 Months |
| Percent of Black Persons in Poverty | 2008-2012 ACS 5-year Estimates. Poverty Status In the Past 12 Months |
|  |  |
| **State-Level Variables** | **Source** |
| Percent registered to Vote | Current Population Survey (CPS) Voting and Registration Supplement (November 2012) (http://www.census.gov/hhes/www/socdemo/voting/publications/p20/2012/tables.html) |
| Percent voted | CPS Voting and Registration Supplement (November 2012) |
| Percent registered to vote- Black | CPS Voting and Registration Supplement (November 2012) |
| Percent voted Black | CPS Voting and Registration Supplement (November 2012) |
| Violent Crime Rate per 100,000 Population | Uniform Crime Reporting Statistics (UCR); US DOJ, FBI; 2010, Crime in the United States, by State, 2010; https://www.fbi.gov/about-us/cjis/ucr/crime-in-the-u.s/2010/crime-in-the-u.s.-2010/violent-crime/violent-crime |
| Aggravated assault rate per 100,000 Population | UCR; US DOJ, FBI; 2010, Crime in the United States, by State, 2010 |
| Property crime rate per 100,000 Population | UCR; US DOJ, FBI; 2010, Crime in the United States, by State, 2010 |
| Burglary rate per 100,000 Population | UCR; US DOJ, FBI; 2010, Crime in the United States, by State, 2010 |
| Larceny-theft rate per 100,000 Population | UCR; US DOJ, FBI; 2010, Crime in the United States, by State, 2010 |
| Motor vehicle theft rate per 100,000 Population | UCR; US DOJ, FBI; 2010, Crime in the United States, by State, 2010 |
| Medicaid (Title XIX) Income Eligibility Pregnant Women (as % of Federal Poverty Level (FPL)) | Kaiser Family Foundation State Health Facts-Medicaid and CHIP-Medicaid/CHIP Eligibility Limits; data as of 2015 |
| Federal MCH Budget per capita | Title V Information System (TVIS) Financial Data for 2010 |
| State MCH Budget percapita | TVIS Financial Data for 2010 |
| Federal MCH Budget per Woman aged 18-44 | TVIS Financial Data for 2010 |
| State MCH Budget per Woman aged 18-44 | TVIS Financial Data for 2010 |
| Black-White Interracial Marriage Rate | ACS 5% sample in Integrated Public Use Microdata Series (IPUMS-USA) |
| Gini Index | 2008-2012 ACS 5-Year Estimates. Gini Index of Income Inequality |
| Non-Hispanic Black Percentage | 2010 Census SF 1 |
| Median Family Income | 2008-2012 ACS 5-Year Estimates. Median Family Income in the Past 12 Months |
| Median Black Family Income | 2008-2012 ACS 5-Year Estimates. Median Family Income in the Past 12 Months |
| Percent of Persons Below Poverty | 2008-2012 ACS 5-Year Estimates. Poverty Status In the Past 12 Months |
| Percent of Blacks Below Poverty | 2008-2012 ACS 5-Year Estimates. Poverty Status In the Past 12 Months |
| Minimum Wage | US Bureau of Labor Statistics, 2010 data |
| Percent of Uninsured Black Females, age 18-64 | Small Area Health Insurance Estimates; Source: U.S. Census Bureau; 2010 data |
| Percent of Uninsured Females, age 18-44 | 2008-2012 ACS 5-Year Estimates. Health Insurance Coverage Status |
