# Supplementary material for: Regional variation in Black infant mortality: The contribution of contextual factors
Source: PLoS One. 2020 Aug 11;15(8):e0237314. doi: 10.1371/journal.pone.0237314 (PMC7418975; doi:10.1371/journal.pone.0237314)
Supplement: S2 Appendix — (DOCX) [file pone.0237314.s002.docx]

**S2 Appendix: Data Source and Notes for All 16 Variables in Final Model.**

| **Variable** | **Level** | **Year** | **Data Source** | **Notes** |
| --- | --- | --- | --- | --- |
| SOCIAL | | | |  |
| Percent non-Hispanic Black Population | state | 2010 | Census Summary File 1 (SF1) | Percent of entire population that identified as non-Hispanic Black. |
| Black-White marriage rate | state | 2008-2012 | American Community Survey (ACS) 5% sample in Integrated Public Use Microdata Series (IPUMS-USA) | Percent of all married non-Hispanic Black individuals with non-Hispanic White spouses. |
| Black incarceration rate (per 100,000 in adult population) | state | 2010 | Census SF1 calculated by prisonpolicy.org | Number of people incarcerated per 100,000 people in that racial/ethnic group): calculated by prisonpolicy.org for the 50 states and was based on 2010 Census data. Incarcerated populations were from all types of correctional facilities in a state (including federal and state prisons, local jails, halfway houses etc.) We calculated the Black incarceration rate for the District of Columbia (D.C.)based on the institutionalized population by race (one-race Black) from the 2010 census for consistency. |
| Percent of voting age population casting votes for 2008 presidential electors | state | 2008 | Current Population Survey (CPS) | Percent of the citizen voting age population (over 18 years of age) that cast a vote in the 2008 presidential election for all races was utilized as the numbers were too small for some states to look at Black-specific rates. The 2008 presidential election was used over the 2010 election (voting for House of Representatives) because D.C. is not included in the 2010 calculation. |
| Segregation Index | county | 2010 | Census SF1 | The segregation index was calculated based on dissimilarity and isolation. Each index was calculated at the county level with the subdivision of census tracts. Both range from 0 to 1. For dissimilarity, 0 is complete integration and 1 is complete segregation and for isolation (minority-weighted average of the minority proportion in each area), a value closer to 1 indicates higher segregation. Commonly a cutoff of 0.6 is considered high for both measures, so we categorized counties as having both dissimilarity and isolation <0.6, counties where one measure was ≥0.6 and counties where both measures were ≥ 0.6. |
| ECONOMIC | | | |  |
| Index of Concentration at the Extremes (ICE) based on Black only Income | county | 2008-2012 | ACS Summary File | The Black index of concentration at the extremes (ICE) based on income: a measure of spatial social polarization. A value of -1 means that all of the population is concentrated in the most deprived (or poor) group and a value of 1 means that all of the population is concentrated in the most privileged (or affluent) group. We utilized the household income at the county level for those who identified as “Black or African-American alone” for their race on the 2008-2012 ACS. The ICE was equal to the number of Black affluent people less the number of poor people over the total Black population. The cutoffs for affluence and poor were utilized in previous studies and were a household income greater than $100,000 and less than $25,000 respectively. |
| Black Civilian Unemployment Rate | county | 2008-2012 | ACS Summary File | Percent unemployed among Black civilians in the labor force. |
| ENVIRONMENT | | | |  |
| Daily Fine Particulate Matter | county | 2010 | 2010 Average Daily Fine Particulate Matter ( PM 2.5) (monitor and modeled) is from the Centers for Disease Control and Prevention Wonder online database | Daily fine particulate matter level: average daily fine particulate matter (PM 2.5) at the county-level was from CDC Wonder’s Online database, based on US EPA AQS PM2.5 in-situ data and NASA MODIS aerosol optical depth remotely sensed data. |
| Grocery Stores per 1,000 population in 2009 | county | 2009 | 2012 USDA ERS Data Download | The USDA calculated the number of grocery stores per 1,000 population in 2009 based on data from the Census Bureau’s County Business patterns and includes establishments generally known as supermarkets and smaller grocery stores (convenience stores and large general merchandise stores such as supercenters were not included). |
| Housing Unit Vacancy Percent | county | 2010 | 2010 Census Redistricting Data Occupied Housing Units | Percent of housing units (independent living quarters) that are unoccupied. |
| NCHS Rural Urban Classification | county | 2006 | National Center for Health Statistics-NCHS Rural Urban Classification (2006) | The 2006 National Center for Health Statistics’ rural-urban classification of counties was utilized and split into three categories (large urban, small and medium urban, and rural counties). |
| HEALTH | | | |  |
| Medicaid eligibility for pregnant women as % federal poverty level (FPL) | state | 2010-2011 | Henry J. Kaiser Family Foundation (KFF) Annual Findings Of A 50-State Survey Of Eligibility Rules, Enrollment and Renewal Procedures, And Cost Sharing Practices in Medicaid and CHIP, 2010-2011 | Based on eligibility thresholds established by state Medicaid programs. |
| Maternal and child health budget per person/capita | state | 2010 | Title V Information System | Calculated based on the 2010 annual state budget figures from the Title V information systems (included obligated balance, total state funds, local MCH funds, other funds and program income (no federal allocation included)) and total state population from the 2010 Census. |
| Certified Nurse Midwives per 100,000 women ages 15-44 years | county | 2011 | Data from Association of Certified Nurse Midwives (population from 2010 Census SF1) | Based on data from the Association of Certified Nurse Midwives (2011) and the denominator of women of reproductive age came from the 2010 Census. |
| Obstetricians/Gynecologists per 100,000 women ages 15-44 years | county | 2011 | Data from American Medical Association Physician Masterfile (population from 2010 Census SF1) | Based on data from the American Medical Association Physician Masterfile (2011) and the denominator of women of reproductive age came from the 2010 Census. |
| Percent Uninsured Females ages 18-44 years | county | 2008-2012 | ACS Summary File | Based on the 2008-2012 ACS, which had both the overall and uninsured female population by age. |
